# Role of anti-diabetic drugs as therapeutic agents in Alzheimer's disease

**DOI:** 10.17179/excli2015-252

**Published:** 2015-05-19

**Authors:** Syed Mohd. Danish Rizvi, Sibhghatulla Shaikh, Shah Mohammad Abbas Waseem, Shazi Shakil, Adel M. Abuzenadah, Deboshree Biswas, Shams Tabrez, Ghulam Md. Ashraf, Mohammad Amjad Kamal

**Affiliations:** 1 Department of Biosciences, Integral University, Lucknow, India; 2 Department of Physiology, Integral Institute of Medical Sciences & Research, Integral University, Lucknow, India; 3 Center of Innovation in Personalized Medicine, Faculty of Applied Medical Sciences,King Abdulaziz University, Jeddah, Saudi Arabia; 4 King Fahd Medical Research Center, King Abdulaziz University, Jeddah, Saudi Arabia; 5 Enzymoic, 7 Peterlee Pl, Hebersham, NSW 2770, Australia

**Keywords:** Alzheimer's disease, anti-diabetic drugs, amyloid beta, beta-secretase, sodium glucose, co-transporters, Type 2 Diabetes Mellitus

## Abstract

Recent data have suggested a strong possible link between Type 2 Diabetes Mellitus and Alzheimer's disease (AD), although exact mechanisms linking the two are still a matter of research and debate. Interestingly, both are diseases with high incidence and prevalence in later years of life. The link appears so strong that some scientists use Alzheimer's and Type 3 Diabetes interchangeably. In depth study of recent data suggests that the anti diabetic drugs not only have possible role in treatment of Alzheimer's but may also arrest the declining cognitive functions associated with it. The present review gives an insight into the possible links, existing therapeutics and clinical trials of anti diabetic drugs in patients suffering from AD primarily or as co-morbidity. It may be concluded that the possible beneficial effects and usefulness of the current anti diabetic drugs in AD cannot be neglected and further research is required to achieve positive results. Currently, several drug trials are in progress to give conclusive evidence based data.

## Abbreviations

AD = Alzheimer's disease; T2DM = Type2 Diabetes Mellitus; ADD = Antidiabetic drugs; 

ADDLs = Aβ-Derived Diffusible Ligands; Aβ = Amyloid β; BBB = Blood brain barrier; 

AMPK = AMP-activated protein kinase; CNS = Central nervous system; BACE-1 = β-secretase; 

PPARγ = Peroxisome proliferator activated receptor gamma; GLP-1 = Glucagon-like peptide-1; 

DPP-4 = Dipeptidyl peptidase-4; SAMP8 = Senescence-accelerated prone; 

SGLTs = Sodium glucose co-transporters

## Introduction

One of the most common causes of progressive dementia in elderly population worldwide is Alzheimer's disease (AD). Exact pathophysiological processes resulting in progressive decline of cognitive functions still remain unclear (Kochanek et al., 2011[54]). 

**Figure 1:**
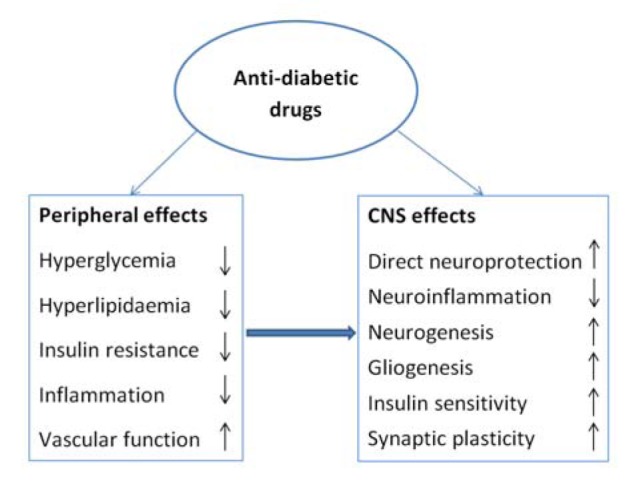
Schematic representation of anti-diabetic drugs as therapeutic agents in neurological disorders

It is fascinating to know that pancreas which is highly innervated organ shares some similarities with brain at molecular (mainly transcriptome and proteome) levels (Allam et al., 2006[5]). Many studies worldwide have shown that Type 2 diabetes mellitus (T2DM) is a risk factor for AD (Whitmer et al., 2008[97]; Biessels et al., 2008[10]; Messier and Gagnon, 2009[63]). Recent epidemiological, clinical and pathological data suggests possible link between T2DM and AD (Akter et al., 2011[4]; Priyadarshini et al., 2012[81]; Park, 2011[74]). AD and T2DM share several molecular processes suggesting possible common pathophysiology. Insulin dysfunction and hyperglycemia in diabetes possibly effects synaptic plasticity, learning and memory which may lead to AD (Akter et al., 2011[4]; Exalto et al., 2012[27]). Abnormalities in various biochemical and physiological pathways involved in cell growth, cell differentiation, cellular repair mechanisms, energy metabolism and glucose utilization could also give rise to AD (Li and Holscher, 2007[60]). In the scenario of ever increasing burden of progressive debilitating diseases like DM and AD with possible common links the silver lining appears to be that “drugs used in treatment of one could effectively be used in the treatment of linked disease”. This novel idea holds true for other diseases sharing common pathophysiological processes. To the best of our knowledge, very few reviews are presented based on this idea. The present communication will focus on various trials of 'Antidiabetic' drugs (ADD), acting as suitable candidates in the treatment of both Diabetes as well as AD. Schematic representation of anti-diabetic drugs as therapeutic agents in neurological disorders has been shown in Figure 1(Fig. 1).

## Anti-Diabetic Drugs against AD

### Insulin

Neuronal cell loss, neurofibrillary tangle (insoluble twisted fibers consisting primarily of the altered Tau protein) deposition inside cell, formation of Amyloid β (Aβ) (39-to-43 peptide originating from the sequential enzymatic cleavage of the larger trans-membrane protein called Amyloid Precursor Protein) plaque in the spaces among neurons and in the walls of blood vessels are microscopic features of AD (Wisniewski and Frackowiak, 1998[98]). Accumulation of Aβ is implicated in the pathogenic cascade (Hardy and Allsop, 1991[38]). In the last decade many observations confirm that small Aβ oligomers (Aβ-Derived Diffusible Ligands (ADDLs)) aggregates are more dangerous and toxic as compared to large aggregates (Picone et al., 2009[78]). The accumulation of these aggregates triggers a variety of responses detrimental to neuron plasticity and memory viz oxidative stress, loss of spines and receptors (De Felice et al., 2009[21]). 

Many researches since early 21st century have highlighted the possible links between AD and insulin signaling (chiefly insulin resistance) associated with T2DM (Ronnemaa et al., 2008[86]; Di Carlo et al., 2010[22]). The link appears so strong that AD is often considered neuroendocrine disorder referred as “Diabetes Type 3” or “Brain Diabetes” (Steen et al., 2005[93]). It has been reported (Zhao et al., 2008[100]) that insulin protects cultured rat neurons against Aβ induced toxicity. It has been shown experimentally that Aβ competes with insulin for the receptor binding thereby decreasing receptor auto phosphorylation (Carrotta et al., 2006[12]). Insulin reduces toxic effects of Aβ by inhibiting fibrillar growth (Rensink et al., 2004[83]). Due to their ability to regulate the mutual binding site abundance Insulin and ADDLs maintain competitive balance between synapse survival and degeneration. Thus with age related progressive decline in brain insulin signaling equilibrium is tilted in favor of ADDLs (De Felice et al., 2009[21]). Newer drugs with ability to maintain the equilibrium could revolutionize the existing treatments available for Alzheimer’s and progressive cognitive function decline. 

There is substantive data available which gives insight into the possible protective role of insulin against Aβ toxicity at the molecular level. Insulin influences the cell viability by inhibiting the pro apoptotic pathways (Di Carlo et al., 2010[22]). Deposition of Amyloid fibrils in pancreatic islets results in oxidative stress which in turn up regulates apoptosis of β cells (Allam et al., 2006[5]). In contrast insulin is shown to reduce oxidative stress by activating serine-threonine kinase Akt signaling pathway which in turn influences several proteins involved in apoptosis; survival is also enhanced by insulin by shuttling Akt into various sub cellular compartments (Lee et al., 2009[59]; Picone et al., 2011[79]). Due to aforementioned evidences and researches it is clear that decrease in insulin signaling with age could lead to AD and this knowledge in turn could be utilized to use insulin as therapeutics in AD. In fact many trials have been done in the past to completely / partially arrest or slow the progression of AD using insulin but the conventional methods available for administration of insulin in diabetics have potential risks in the AD patients, thus alternate routes are being explored. Intra nasal Insulin proved beneficial in dementia associated with AD, probably by changing Aβ 42 levels and Tau protein-to-Aβ 42 ratios in cerebrospinal fluid (Craft et al., 2012[19]). But the treatment is associated with certain side effects like irritation, nasal mucosa damage (Nolte et al., 1990[73]) and increase in blood pressure (Cernea and Raz, 2006[15]). The associated side effects can be reduced by delivering insulin directly to the brain which requires overcoming of blood brain barrier (BBB).

## Insulin Sensitizers

### Biguanides (Metformin)

**Figure 2:**
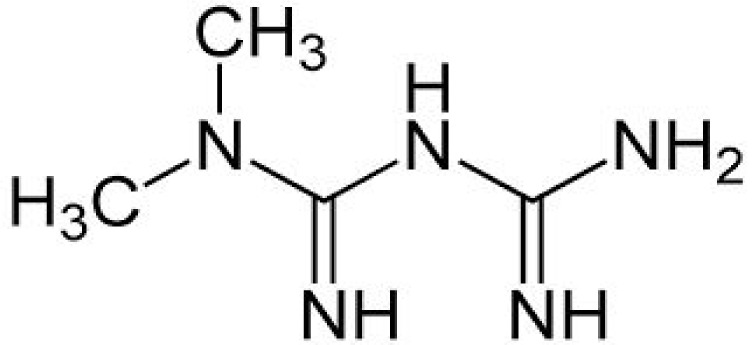
Structure of Metformin

Metformin (N, N dimethyl imidodi carbonimidic diamide) (Figure 2(Fig. 2)) is an orally active biguanide that has glucose lowering effects (suppress gluconeogenesis in liver), decreases insulin resistance, potentiates insulin action and increases insulin sensitivity of liver and skeletal muscle via AMP-activated protein kinase (AMPK) (Zang et al., 2004[99]; Hilder et al., 2005[42]). AMPK acts as central regulator of lipid and glucose metabolism and upon activation increases insulin sensitivity through interactions with a multiple intracellular targets like Mammalian Target of Rapamycin, p38 Mitogen-Activated Protein Kinases, and Protein Kinase C. Conclusive data on the penetration of metformin and potential effects in human CNS is lacking but studies in rodents have shown that metformin does cross the BBB and activates AMPK in central nervous system (CNS) tissue (Łabuzek et al., 2010[57]; Nath et al., 2009[71]). Experimental models have shown that Metformin provides neuroprotection via variety of mechanisms like reducing phosphorylation of tau protein in cortical neurons (Kickstein et al., 2010[51]), prevention of apoptotic cell death in primary neurons (El-Mir et al., 2008[25]), by improving oxygen-glucose deprivation induced neuronal injury and thus enhancing survival of neurons (Mielke et al., 2006[64]). Similarly, various other studies have talked about the potential benefits of treatment with metformin. Hwang and colleagues (2010[46]) reported that treatment of Zucker diabetic fatty rat with metformin normalized the reduction of cell proliferation and neuroblast differentiation in hippocampal dentate gyrus. Gupta et al., (2011[36]) reported an increase in insulin action which in turn prevented molecular and pathological characteristics associated with AD in a cell cultured model of insulin resistance (induced by chronic exposure of mouse neuroblastoma cell line, Neuro-2a (N2A) to insulin) upon exposure to metformin. Increased neuronal viability was reported in an *in vitro* model of ischemia after treatment with metformin (Mielke et al., 2006[64]). Thus, metformin may prove beneficial in reducing neuropathy and neuronal cell injury associated with hyperglycemia in diabetes. Interestingly, a Taiwanese clinical study conducted in human subjects found that the use of metformin significantly decreases the risk of dementia (Hsu et al., 2011[45]). Further clinical trials based on the above information and experimental designs could prove to be beneficial.

Effects of metformin on Beta-site amyloid precursor protein cleaving enzyme-1/β-secretase (BACE-1) in experimental models have also generated interest of researchers. BACE-1 is the major protease of amyloid precursor protein pathway which generates and accumulates Aβ in brain (levels directly correlate with pathogenesis of AD). Novel inhibitors by directly targeting BACE-1 could reduce levels of Aβ in brain (Kirpichnikov et al., 2002[52]; Knowler et al., 2002[53]). Interestingly, treatment with the ADD metformin decreases BACE1 protein expression and activity, thereby reducing it's cleavage product Aβ (Gupta et al., 2011[36]). Similarly, Chen and colleagues (2009[16]) observed low Aβ production in culture medium in presence of insulin (reduction could further be enhanced by addition of metformin) as compared to increased BACE1 mRNA levels, protein levels and A*β* production in the culture devoid of insulin. 

Other area of interest is role of metformin on Midline1 protein complex, which is implicated in translation of various protein complexes involved in neurodegeneration (Aranda-Orgilles et al., 2011[7]; Krauss et al., 2013[56]). Metformin interferes with assembly of Midline1 protein complex thereby reducing translation of BACE 1 mRNA which in turn decreases Aβ production and tau-phosphorylation (Kickstein et al., 2010[51]). Thus, Metformin is being explored as suitable drug in treatment of AD since it targets both Aβ production and tau-phosphorylation which are hallmarks of the disease. Moreover it appears to be less harmful even if it suppresses the translation of multiple mRNAs regulated by Midline1, as it is already being used as ADD.

### Thiazolidinediones (Rosiglitazone and Pioglitazone)

Thiazolidinediones are stimulators of peroxisome proliferator activated receptor gamma (PPARγ) and apart from having anti diabetic potentials also appear to have anti-amyloidogenic, anti-inflammatory, and insulin sensitizing effects, thus signifying the role in delaying and reducing the risk of neurodegeneration (Heneka et al., 2001[40]). Neuroprotection is provided by lowering peripheral insulin and enhancing insulin sensitivity, reducing inflammation and Aβ accumulation and also by increasing cerebral blood flow (Sato et al., 2011[87]; Landreth, 2007[58]). Potential role in treatment of AD include reductions in inflammatory cytokines (Heneka et al., 2005[41]), oxidative stress (Nicolakakis et al., 2008[72]), Aβ deposits (Heneka et al., 2005[41]), glial activation (Nicolakakis et al., 2008[72]), tau phosphorylation (To et al., 2011[95]), and glucocorticoid signaling (Escribano et al., 2009[26]; Garcia-Bueno et al., 2005[31]). Thiazolidinediones also improve cognition independent of their effect on glucose regulation as shown in the Tg2576 model of AD with diabetes (Rodriguez-Rivera et al., 2011[85]). 

Rosiglitazone have protective role against neuronal insulin resistance induced by Aβ oligomers (De Felice et al., 2009[21]). They are shown to increase brain Insulin-degrading enzyme levels in an animal model of AD (Pedersen et al., 2006[75]). Watson and colleagues (2005[96]) conducted a study on patients with mild to moderate AD and showed that in patients without ApoE ε4 allele there was improvement in cognition and modulation of A*β* levels in cerebral spinal fluid while patients with ApoE ε4 did not respond to the drug (Rosiglitazone) and showed no improvement. Possible explanation could be role of drug on mitochondria in the brain thereby increasing their metabolic efficiency and number (Craft et al., 2006[19]). Encouraging preliminary data based on smaller pilot studies led to sponsoring of phase 3 studies of rosiglitazone in AD but unfortunately larger trials could not replicate positive findings (Harrington et al., 2009[39]).

**Figure 3:**
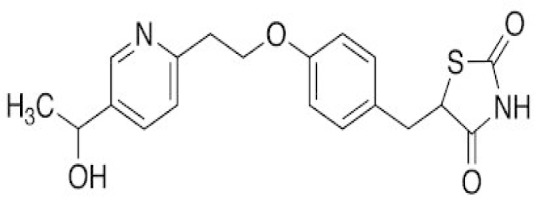
Structure of Pioglitazone

The possible role of oxidative stress in the pathogenesis of AD has been highlighted recently (Calissano et al., 2009[11]). Activation of microglia in AD results in production of large quantities of free radicals including peroxynitrite formed due to interaction of NO with oxygen (Murphy, 2000[70]). Ji and colleagues (2010[49]) showed that the levels of NO produced by proinflammatory mediators in cultured microglial cells can be reduced by treatment with PPAR-γ agonists. Similarly, Heneka and colleagues (2005[41]) concluded that iNOS expression in cerebellum can be suppressed by troglitazone and pioglitazone (Figure 3(Fig. 3)) thus protecting granule cells against lipopolysaccharide/ interferon-γ insult. Pioglitazone are thought to act as scavengers of free radicals and peroxynitrate in the sera of AD patients (Ceconi et al., 2007[13]). Gray (2012[34]) discussed the role of pioglitazone as xeno protective agent in vitro. In another study, the triple transgenic mouse model of AD with accelerated Aß deposition and tau pathology was treated with the pioglitazone (18 mg/Kg body weight/day). After four months, treated animals exhibited improved learning on the active avoidance task, decreased hippocampal Aß and tau deposits, and enhanced short- and long-term plasticity. Surprisingly, peripheral glucose concentrations were not altered suggesting that pioglitazone acted, at least in part, through mechanisms other than glucose regulation (Murphy et al., 2012[69]).

## Insulin Secretagogues

### Third-Generation Sulfonylureas (Glimepiride)

**Figure 4:**
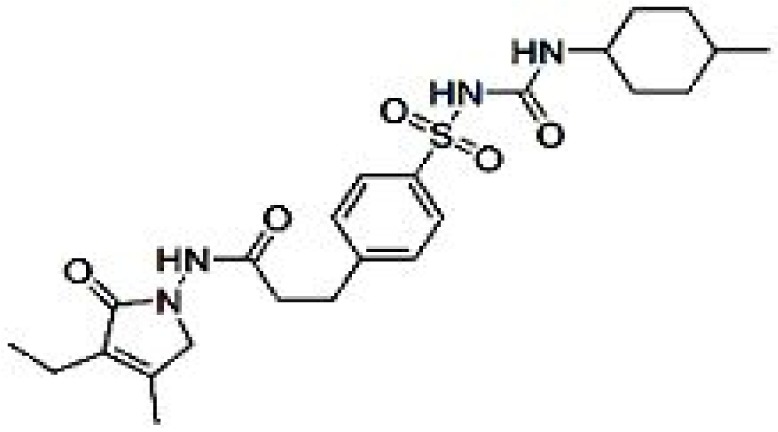
Structure of Glimepiride

*Glimepiride* (Figure 4(Fig. 4)) binds to the sulfonylurea receptor SUR1 present on the pancreatic cells membrane which in turns stimulates insulin secretion by closing potassium channel (Farret et al., 2005[29]). It is also reported to have other extra pancreatic effects like increasing glucose uptake, stimulating the release of GPI-anchored proteins and activating PPARγ (Fukuen et al., 2005[30]). Reportedly it docks to PPARγ and exhibits PPARγ agonistic activity in cell-based transactivation assay (Scarsi et al., 2007[89]). It also enhances the interaction of PPARγ with cofactors and up regulates the expressions of PPARγ target genes, including aP2 and leptin (Fukuen et al., 2005[30]). PPARγ activation is demonstrated to decrease levels of A*β* and senile plaques which improves the cognitive function in AD patients (Jiang et al., 2008[50]). Due to similarity in structure with BACE-1 inhibitor sulfonamide it is indicated that glimepiride possess BACE-1 inhibition properties and thus down regulates A*β*. Apart from glimepiride another sulfonylurea drug glipizide, enhances cognitive function of T2DM patients (Testa and Simonson, 1998[94]). Results of study conducted by Liu and colleagues (2013[61]) show reduce production of A*β* in primary cortical neurons which were attributed to suppression of BACE1 activity and down regulation of BACE1 mRNA and protein expression. The result appears more promising and highlights the future role of glimepiride in treatment of AD associated with diabetes in lieu of fact that hyperglycemia enhances A*β* 40 production and glimepiride significantly lowers hyperglycemia induced A*β* 40 production.

## Peptide Analogs

Insulin like metabolic hormones like Glucagon-like peptide-1 (GLP-1) and Amylin A (structurally unrelated to insulin and binding to independent receptors in brain) provide another opportunity to restore insulin signaling in the AD brain. Analogs of these two hormones are approved or are in the process of regulatory approval in the USA. The signaling pathways activated by them converge and facilitate insulin signaling. They readily cross the BBB. Since they do not cause significant hypoglycemia (as opposed to insulin), thus can be safely administered at relatively high doses peripherally. 

The hormone GLP-1 (secreted by intestinal L cells in response to a meal) lowers glucose by various mechanisms like increasing insulin secretion, decreasing glucagon secretion, delayed gastric emptying, increased satiety, and increased insulin sensitivity in multiple tissues (American Diabetes Association, 2013[6]). The GLP-1 receptor is nearly ubiquitous and is widely expressed in the brain. Since dipeptidyl peptidase-4 (DPP-4) rapidly degrades endogenous GLP-1 thus for treatment of T2DM injectable analogs of GLP-1 (exenatide and liraglutide) with prolonged half-lives have been developed and approved. Multiple oral inhibitors of DPP-4 that indirectly augment endogenous GLP-1 levels have also been approved for T2DM. Studies conducted in cell culture and mouse models of AD using GLP1 have shown encouraging results (McClean et al., 2011[62]). These agents provide protection against oxidative injury and also cause synaptogenesis and neurogenesis thus acting as growth factor in brain. There were two pilot studies conducted of GLP-1 analogs for AD (3-year 230-patient randomized trial using exenatide sponsored by National Institute on Aging (Clinical Trials identifier: NCT01255163) and a 12-month, 200-patient trial of liraglutide conducted by the Imperial College London (Clinical Trials identifier: NCT01843075)). In AD mouse models, GLP-1 agonists are shown to improve memory behaviors, reduce levels of AD pathologic markers (oligomeric A*β* and A*β* plaque load) and decrease activation of microglia.

Amylin (islet amyloid polypeptide), is a peptide hormone co-secreted with insulin from β-cells of pancreas (Mitsukawa et al., 1990[66]) and lowers blood glucose levels (like GLP-1) through delayed gastric emptying, decreased glucagon secretion, and increased satiety (Gedulin et al., 1997[32]). Utility of endogenous amylin is limited due to its propensity to aggregate and form amylin oligomers and plaques. Recently in Alzheimer's brain tissue abnormal Amylin has been recognized. Jackson and colleagues (2013[47]) reported oligomeric and plaque-like accumulations of Amylin in brain parenchyma and cerebral vasculature of diabetics as well as non diabetic AD patients. Interestingly, these plaques though independent were sometimes co localized with A*β* plaques. Recently, in a rodent model of accelerated aging an amylin agonist has been investigated as a potential therapeutic approach for the treatment of AD (Adler et al., 2014[2]). 

Amylin readily crosses the BBB (Banks et al., 1995[9]), and its receptors are distributed widely throughout the brain (area postrema, nucleus of the solitary tract, parabrachial nucleus, amygdala, hypothalamus, nucleus accumbens, and dorsal raphe (Sexton et al., 1994[91])) thus, it is thought to have widespread implications in variety of CNS functions (like memory, mood, anxiety and satiety). Amylin interacts with signaling cascade in peripheral tissue and CNS which activates several downstream targets of insulin like signal transducer and activator of transcription 3, AMP-activated protein kinase, and Akt cascades involved in cellular metabolism and survival (Moon et al., 2011[67]; Mihaylova and Shaw, 2011[65]). Amylin is also a known modulator of the extracellular-signal-regulated kinases/mitogen-activated protein Kinase pathway which effects satiety and is thought to play a role in synaptic plasticity and memory consolidation in the hippocampus (Potes et al., 2012[80]). Adler and colleagues (2014[2]) reported an improved memory performance in the novel object recognition task and modulation of synapses, decrease oxidative stress and inflammatory markers in the hippocampus of mice following chronic infusion of pramlintide (soluble, non aggregating synthetic analog of amylin), in mice. Morley and colleagues (2012[68]) demonstrated the direct effects of amylin on cognition and AD pathogenesis by showing the effects of pramlintide on memory in senescence-accelerated prone (SAMP8) mouse (model of sporadic AD), the results indicated reduced expression of HO-1 (cellular stress protein that is activated during high oxidative stress and inflammatory states). Expression of HO-1 is increased in the hippocampus and cerebral cortex of AD brains (Schipper et al., 1995[90]) and age-dependant increase in the SAMP8 mouse (Cuesta et al., 2010[20]). Pramlintide decreases HNE which is a byproduct of lipid peroxidation and act as early stress marker in AD brain (Sayre et al., 1997[20]), it also reduces COX-2 (marker of inflammation) which increases in AD as well as in brain with aging (Ho et al., 1998[43]). Other studies have also reported beneficial role of pramlintide in oxidative stress and inflammation in peripheral tissues (Ceriello et al., 2005[14]). It is worth noting that Pramlintide causes minimal hypoglycemia, and its excellent safety profile and tolerability are appealing for the study and treatment of AD.

Injectable Glucagon-like peptide analogs and agonists

GLP-1 is an endogenous 30-amino acid peptide hormone. Neuroprotective role GLP-1 analogues have been shown in various *in vitro* and *in vivo* studies. GLP-1 receptors are expressed in the hippocampal area (important site of neurogenesis in adults and role in cognition and memory). The neuroprotective functions of GLP-1 analogues can be realized only if they cross BBB and bind to their receptors. In fact many novel long-lasting GLP-1 receptor agonists have been developed in recent years like Exendin-4 (Byetta), Liraglutide (Victoza) and lixisenatide (Lyxumia) (Christensen et al., 2011[17]; Faludi et al., 2009[28]).

GLP-1 acting as a growth factor in the brain has been shown to induce neurite outgrowth and protect against oxidative injury in cultured neuronal cells (Perry et al., 2007[76]). Increased neurite growth and improvement in learning was shown in mice which over expressed GLP-1 receptors in hippocampus (During et al., 2003[23]). Abbas and colleagues (2009[1]) reported impairments in long-term potentiation of synaptic transmission in the hippocampus and learning of new tasks upon deletion of GLP-1 receptor. Widespread effects of GLP-1 and analogs have been reported. Reductions in endogenous levels of A*β* in the brain have been reported upon use of GLP-1 and exendin-4 (Perry et al., 2003[77]). Proliferation of neuronal progenitor cells in the brain of mice is induced by GLP-1 analogs (During et al., 2003[23]). It is interesting to note that AD patients could benefit due to increase in cell proliferation which facilitates repair of neuronal networks in cortical tissue (Greenberg and Jin, 2006[35]). Gengler and colleagues (2012[33]) reported long-term potentiation in the hippocampus when Val(8) GLP-1 was injected intraperitoneally for 3 weeks. Reported results are encouraging and GLP-1 analogs could prove beneficial in improving cognition, neuronal communication and regeneration of neurons in AD patients (Holscher and Li, 2010[44]). 

Peripheral injection of liraglutide increases neuronal progenitor cell proliferation and neurogenesis in the brain (Hamilton et al., 2011[37]). It is a known fact that detrimental long-term effects of inflammation are one of the important underlying mechanisms of neurodegeneration in AD (Aisen, 2002[3]). Jankowsky and colleagues (2001[48]) injected liraglutide (which crosses the BBB) intraperitoneally in mouse model of AD for 8 weeks and demonstrated the improvements in learning (object recognition and water maze tasks) which were supported by a significant protection of long-term potentiation and paired-pulse facilitation. In the same model the reduction in synaptic numbers was partly prevented by the drug which in turn improves cognitive functions. The results assume further importance when it was shown that the drug reduces amyloid synthesis. Importantly, in liraglutide treated model inflammatory responses in the brain (evaluated by the number of activated microglia) were less and the number of new neurons in the dentate gyrus was normalized to wild-type control levels.

Agents like dipeptidyl peptidase-4 (DPP-4) inhibitors increase the levels of glucagon-like peptide-1 (GLP-1) and can act as suitable disease modifying agents in the treatment of AD. In a recent study done by Kosaraju (2013[55]), treatment of rat models induced with AD (by intracerebral injection of STZ) with Vildagliptin (DPP-4 inhibitor) showed dose and time-dependent improvements in memory retention and dose-dependent attenuation of A*β*, tau phosphorylation and inflammatory markers thus indicating that with increase in GLP-1 levels there is clearance of A*β* and tau which is beneficial in AD.

## Glycosurics

### SGLT-2 inhibitors (Canagliflozin and Dapagliflozin)

**Figure 5:**
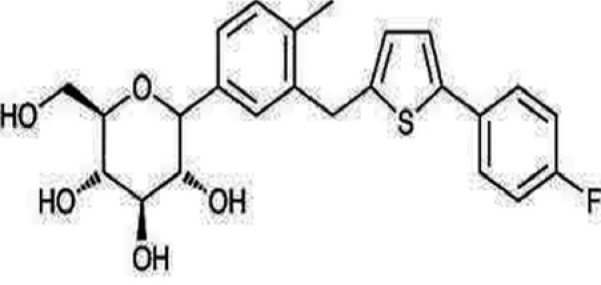
Structure of Canagliflozin

**Figure 6:**
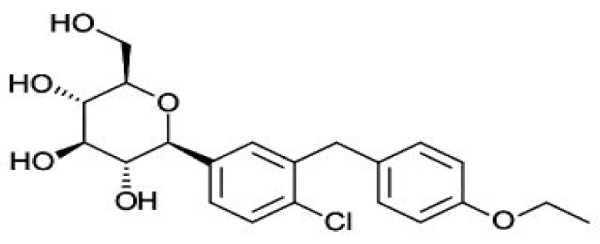
Structure of Dapagliflozin

Sodium glucose co-transporters (SGLTs 1 and 2) cause renal glucose reabsorption (mostly by SGLT-2) (Rajesh et al., 2010[82]). Since SGLT-2 is expressed exclusively in kidney thus its inhibition act as a therapeutic target in T2DM without risk of hypoglycemia since it increases renal excretion of glucose without influencing insulin secretion (Edward and Robert, 2010[24]). In fact, both pre and postprandial blood glucose levels are reduced on acute administration and there is decrease glucotoxicity on chronic administration of SGLT inhibitors (Bakris et al., 2009[8]). Recently, two SGLT-2 inhibitor drugs have been approved by Food and Drug Administration (FDA), namely “Invokana” (chemically known as canagliflozin) (Figure 5(Fig. 5)) and “Forxiga” (chemically known as Dapagliflozin) (Figure 6(Fig. 6)). Recent studies by Rizvi et al. (2014[84]) and Shaikh et al. (2014[92]) have explored the molecular interactions of human brain acetylcholinesterase with these antidiabetic drugs. Results suggest that these might act as inhibitor of acetylcholinesterase and SGLT2. However, further investigations are needed to address the issue regarding their dual inhibitory roles against T2DM and AD.

## Conclusions

Diabetes and AD have traditionally been thought to be independent disorders. However, the results of recent investigations and researches have suggested possible interactions which could lead to effective common treatment regimes. Many clinical trials are underway to test effectiveness of ADD targeting brain insulin signaling in AD patients. The complexity of the task at hand and challenges in improving brain functions including cognition have made the work more interesting specially as far as testing “AD-T2DM hypothesis” is concerned. The results of these trails will give an insight into the mechanisms which connect these two progressive and challenging diseases affecting mankind and will also add new chapters in pharmacotherapy.

## Conflict of interest

The authors confirm that this article content has no conflict of interest.

## Acknowledgements

Sibhghatulla Shaikh is supported by *INSPIRE* grant from Department of Science & Technology (DST), New Delhi, India (Grant Number: IF130056), which is sincerely acknowledged. Shazi Shakil thanks all of the staff of Center of Innovation in Personalized Medicine (CIPM), King Abdulaziz University, Saudi Arabia for continued support.
